# Sensor-to-Image Based Neural Networks: A Reliable Reconstruction Method for Diffuse Optical Imaging of High-Scattering Media

**DOI:** 10.3390/s22239096

**Published:** 2022-11-23

**Authors:** Diannata Rahman Yuliansyah, Min-Chun Pan, Ya-Fen Hsu

**Affiliations:** 1 Department of Mechanical Engineering, National Central University, Taoyuan City 320, Taiwan; 2 Department of Surgery, Landseed International Hospital, Taoyuan City 324, Taiwan

**Keywords:** diffuse optical imaging, image reconstruction, inverse problem, Tikhonov regularization (TR), deep modeling, convolutional neural networks, residual net, skip connection

## Abstract

Imaging tasks today are being increasingly shifted toward deep learning-based solutions. Biomedical imaging problems are no exception toward this tendency. It is appealing to consider deep learning as an alternative to such a complex imaging task. Although research of deep learning-based solutions continues to thrive, challenges still remain that limits the availability of these solutions in clinical practice. Diffuse optical tomography is a particularly challenging field since the problem is both ill-posed and ill-conditioned. To get a reconstructed image, various regularization-based models and procedures have been developed in the last three decades. In this study, a sensor-to-image based neural network for diffuse optical imaging has been developed as an alternative to the existing Tikhonov regularization (TR) method. It also provides a different structure compared to previous neural network approaches. We focus on realizing a complete image reconstruction function approximation (from sensor to image) by combining multiple deep learning architectures known in imaging fields that gives more capability to learn than the fully connected neural networks (FCNN) and/or convolutional neural networks (CNN) architectures. We use the idea of transformation from sensor- to image-domain similarly with AUTOMAP, and use the concept of an encoder, which is to learn a compressed representation of the inputs. Further, a U-net with skip connections to extract features and obtain the contrast image, is proposed and implemented. We designed a branching-like structure of the network that fully supports the ring-scanning measurement system, which means it can deal with various types of experimental data. The output images are obtained by multiplying the contrast images with the background coefficients. Our network is capable of producing attainable performance in both simulation and experiment cases, and is proven to be reliable to reconstruct non-synthesized data. Its apparent superior performance was compared with the results of the TR method and FCNN models. The proposed and implemented model is feasible to localize the inclusions with various conditions. The strategy created in this paper can be a promising alternative solution for clinical breast tumor imaging applications.

## 1. Introduction

Recently, deep learning has emerged as a powerful tool to solve many imaging problems. Promising results have been achieved in various imaging tasks, such as classification [1] and segmentation [2]. Following such success, several methods using deep neural networks have been developed for biomedical image reconstruction. Applications of deep neural networks have been found in problems such as X-ray computed tomography [3] and magnetic resonance imaging [4]. A unified framework called AUTOMAP can perform image reconstruction for multiple problems in a single network [5].

**Figure 1:**
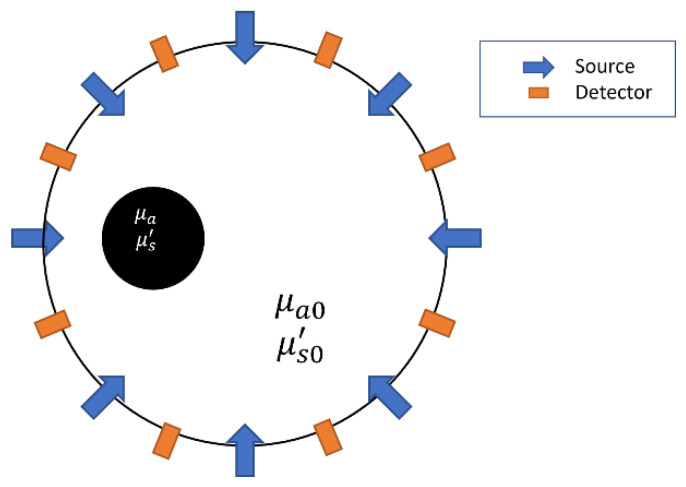
Illustration of optical information measurement for DOT imaging.

Diffuse optical tomography (DOT), or diffuse optical imaging, is a non-invasive technique to evaluate the optical properties of the biological tissue in the diffusive regime by using near-infrared (NIR) light, and particularly can be used to detect breast tumor or other soft tissue lesions [6,7,8], as shown in Figure 1. The quality of the results obtained depends on measurement systems and image reconstruction methods used. The measurement systems based on source types can be classified into continuous wave, time-domain, and frequency domain (FD) systems.

**Figure 2:**
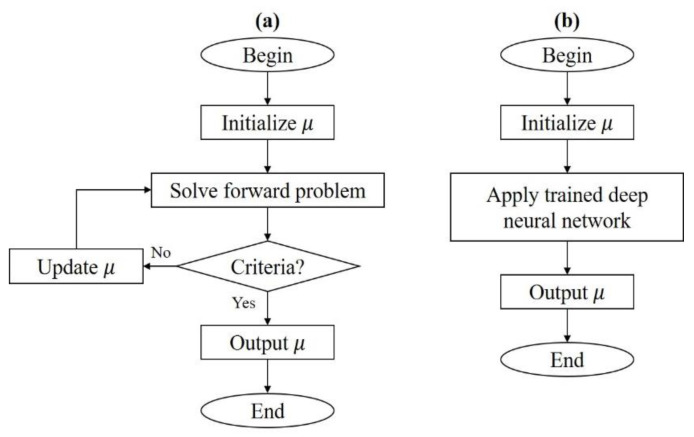
Flowcharts of image reconstruction methods. (**a**) Iterative method and (**b**) deep neural network.

DOT consists of two main problems, i.e., the forward and the inverse problems [9,10]. The forward problem is the computation of photon density given light source information and optical property distribution, while the inverse problem is an attempt to reconstruct optical properties referring to the positions for boundary data given. The inverse problem. or the computation of optical-property image reconstruction in DOT, remains challenging since it is ill-posed and ill-conditioned. Regularization methods are commonly used to mitigate the ill-posedness nature of the problem [11]. To reflect better-quality performance, systems using various wavelengths were developed [12,13]. While these methods can give feasible and sufficient quality results, some unwanted drawbacks still exist. Generally, a single reconstruction process might take much computation time and large storage memory. Based on how the algorithm works, there is a trade-off between resolution and computational cost. One can use a hardware design with multi-modality, more measurement data, and also optimize the geometry by using a finer mesh with a larger amount of nodes, to get high-quality results. However, this demands heavy computational costs when using iterative methods. The forward problem must be solved and the large distribution of the optical properties must be updated at each iteration [10], which might only be updated with small values when the convergence is slow. Thus, an alternative method with a fast and efficient reconstruction process is desirable. Since deep learning based methods are already known to be successful at handling imaging problems, it is probable to implement such a method for DOT. Using deep neural networks as a reconstruction method is pretty straightforward. After being trained, a deep neural network can be given a measured data set as input and the reconstructed image as output can be obtained. Figure 2 shows the flowcharts of iterative methods and deep neural networks.

Some applications of deep learning for DOT problems already exist, although there are only a few similar papers and they are still considered preliminary. Unlike other image reconstruction problems, DOT physics is known to be highly non-linear and ill-posed [10]. A deep learning approach to improve the image reconstruction of a hand-held diffuse optical breast scanner probe was implemented [14]. Recently, a back-propagation neural network (BPNN) has been used for DOT image reconstruction by simulation experiments for training and validation [15]. A study also employed deep learning architecture for 3D DOT image reconstruction, which employed phantom and in-vivo data [16].

When we want to consider a suitable, deep learning based solution for DOT, a certain setup needs to be considered, since one application and another will be different. In a way, we want the method to be capable of flexibility and relevance with the real measurement system. Table 1 lists related works from previous studies. These implementations give promising results with certain setup for the experiments, but there are factors that have not been considered. One that is common between these implementations is that the output produces absorption coefficient values. Commonly, scattering coefficient is also considered in DOT. Adding scattering coefficient values as output may increase the complexity of the neural networks purposed for the reconstruction. Furthermore, these neural networks are implemented case by case, specialized for certain experiment types, and only maintain the architecture. This limits wide-range usage for DOT. It remains challenging to implement neural networks capable of various experiment types, handling large absorption changes, and robust to the deviations of the functional (light-propagation equation) model [16].

Within this study, a deep learning model based on both AUTOMAP and U-net was proposed to reconstruct the absorption and reduced scattering coefficient images of breast-like phantoms. Previous existing implementations commonly applied fully-connected and/or convolutional layers [14,15,16]. Knowing the applied deep learning schemes from the existing applications and the prepared datasets, then we can adopt and implement deep learning based methods for the study. The proposed approach can be an alternative solution to conventional methods that offers simple and straightforward reconstruction process since we only focus on the task with a particular experiment setup. We implement a deep learning based algorithm specifically for resolving the high-scattering-media imaging, or probably breast tumor imaging. The neural networks implemented will be focused to improve reconstruction for the FD ring-scanning device/measurement system [17].

## 2. Methodology

### 2.1. Diffuse Optical Imaging (Forward and Inverse Problems)

As explained previously, DOT consists of forward and inverse problems. The conventional method mainly deals with solving these problems, which is to compute a photon density formula from known optical properties, and vice versa. Highly scattered NIR light propagation in tissues can be described by the diffusion equation. For the FD system it is given by [6,10]

(1) $$\nabla \cdot D\nabla \Phi \left(r,\omega \right)-\left(\mu_{a}-\frac{i\omega }{c}\right)\Phi \left(r,\omega \right)=-S_{0}(r,\omega ),$$

where Φ(r,ω) is the radiance, D is the diffusion coefficient, $\mu_{a}$ is the absorption coefficient, c is the wave speed in the medium, and $S_{0}\left(r,\omega \right)$ is the source term. The diffusion coefficient D is given by

(2) $$D=\frac{1}{3\left[\mu_{s}\left(1-g\right)+\mu_{a}\right]}=\frac{1}{3\left(\mu_{s}^{\prime }+\mu_{a}\right)},$$

where $\mu_{s}$ is the scattering coefficient, *g* is the average cosine of the scattering angle, and $\mu_{s}^{\prime }$ is the reduced scattering coefficient. Equation (1) is a standard boundary-value problem for the spatially varying radiance subject to appropriate boundary data [6,10].

Because of the non-linearity of the inverse problem, we iteratively minimize the data-model misfit difference $\chi^{2}=\sum_{i=1}^{N_{M}}{\left[\Phi_{i}^{C}-\Phi_{i}^{M}\right]}^{2}$ by solving JΔX=ΔΦ, where $\Phi^{M}$ and $\Phi^{C}$ denote the measured and calculated photon density, respectively, $J=\left[\begin{array}{cc} \frac{\partial \Phi^{C}}{\partial D} & \frac{\partial \Phi^{C}}{\partial \mu_{a}} \end{array}\right]$ is the Jacobian matrix, and ΔX denotes $\left[\begin{array}{c} \Delta D\\ \Delta \mu_{a} \end{array}\right]$, the optical coefficients update at each iteration [7].

The process starts by assigning some initial guess for optical properties ($\mu_{a}$ and $\mu_{s}^{\prime }$), solve the forward model to get the corresponding Φ(r), then compare it with the measured Φ(r) to check for some criteria. If those criteria are met, then the computation is stopped and the optical properties are obtained. If they are not, then update the optical properties and repeat the process until those criteria are met.

### 2.2. Datasets

**Figure 3:**
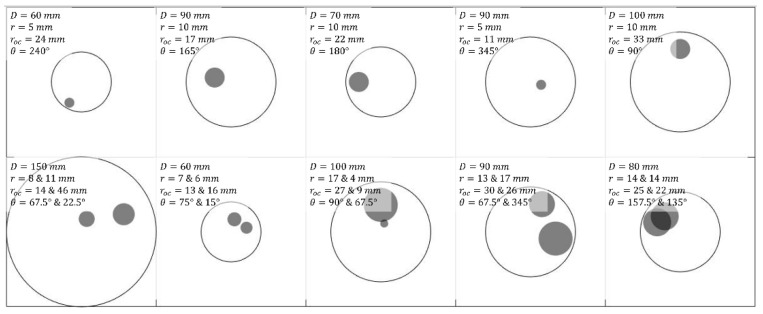
Some examples of training phantom designs with varied parameters, where *D*, *r*, *r_oc_* and *θ* denote phantom diameter, inclusion radius, off-center distance of inclusion(s) and orientation of inclusion(s), respectively.

A training dataset including 10,000 samples was created with different designations of phantom cases. The parameters of these phantom cases are specified in Table 2 based on various properties, and all samples were chosen by a random uniform distribution of the parameter selection. For each sample, each of the parameters that define a sample is chosen from the range associated with it. For example, picking one sample from all samples with 2 inclusions (with total of 5500 samples), each of the inclusions will be placed at different location over the phantom. Both inclusions could have diameters ranging between 4–17 mm. This range along with the phantom sized between 60–150 mm in diameter are adjusted based on measurement device in practice [17]. The range for coefficients are also adjusted based on existing known range for breast tissues from clinical cases [18]. We employed the phantom profile with circular shape as it was the most frequently considered in previous studies [17,19,20,21]. The use this shape was kept as a starting point within this study. The image output will be kept the same as will be explained later. Examples of phantom cases are illustrated in Figure 3.

For each sample, the input data are in the form of N_s_ (source locations) × N_d_ (detector locations) × 2 (log magnitude and phase lag), which are all floating-point values. The output data are in the form of a rectangular grid of size 64 × 64, which stores floating-point values of absorption or scattering coefficients. These values have been interpolated from the original data of 3169 nodes. Although the phantom sizes are different based on the diameters of each one of them, the resulted image will always be the same 64 × 64 matrix. We only use the diameter for input and visualizing purposes. The ground truth generated by constructing the image of the breast-like phantom and the tumors in the rectangular grid, computed directly from the parameters given in Table 2 with the shape of simple non-distorted circles.

Meanwhile, a test dataset was created by gathering experimental data that had been obtained previously through phantom test at our laboratory. Here, we do not set a mixture of experimental data against training data as we do for validation data, since we only have limited samples available for experiment compared to simulation. We realize the limitations of considering experimental data for such complicated inverse problem in deep learning, since these kinds of data are noisy and potentially could have some errors in the process that caused by various factors. It is desirable to consider more data, but it will require an extensive amount of time and resources. We gather few experiment data readily available and we will compare the results between the simulation training data and the experimental testing data in this study.

A total of 10 samples were chosen, each with 16 source locations and 15 detections. To avoid data discrepancy between the input data from simulation and experiment, it is necessary to calibrate the input data from experiments before testing them on the deep learning model. The calibrated data were computed accordingly provided that both homogeneous and inhomogeneous data are present from the same phantom case. We may want to consider other potential cases of experimental data. These may not be covered since only few data gathered, but for the sake of the starting research for alternative method and the complexity of the problem applied with deep learning [15,16], it should be enough to have some analysis on the capability of the alternative method.

### 2.3. Network Architecture

One can consider AUTOMAP as a general framework when we try to approach an image reconstruction method with different domains between input and output (from sensor to image) [5]. The non-linearity of DOT as an inverse scattering problem brings the motivation to consider a deep learning solution. Linear problems such as MRI and CT are well defined problems, so using neural networks may not be necessarily recommended. Remember that neural networks are a form of approximation for an analytical function [22]. The cost to build the neural networks depends on the number of trainable parameters. We already know the examples of FCNN added with CNN previously. Using FCNN can cause the number of trainable parameters increasingly huge along with input and output size. From Table 1, most of the parameters are from FCNN. Therefore, we may want to consider one or only few fully-connected layers to transform between the different dimensions of the input (sensor) and the output (image). CNN can work to filter the initial reconstructed image to improve the result. So, we can have FCNN added with CNN at least in order to be useful as a reconstruction method as have been successfully implemented previously. The concept of AUTOMAP is already applied in such implementations, since DOT is to map between sensor data to reconstructed image. In our study, we want to consider factors mentioned before that have not yet been covered in previous studies. We want to build a “full feature” deep learning solution that is capable of handling various data that can be obtained from the FD ring-scanning device and gives accurate reconstructed image.

To achieve the desired architecture, we introduce an abstraction in the form of blocks of basic structures (hidden layers) to be added, or purposed for specific functionality or transformation to the whole network. Then, these blocks have their own architectures to be defined.

**Figure 4:**
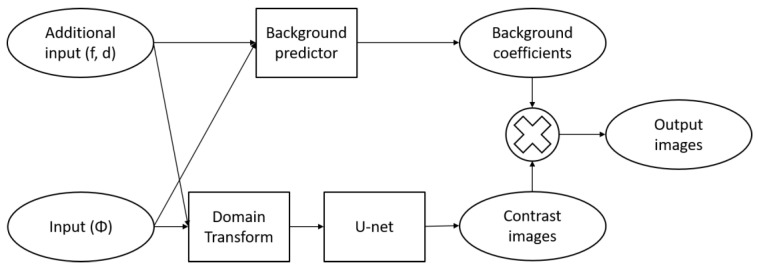
Block diagram of the proposed model, where Φ, f and d denote input radiance, FD frequency and phantom diameter, respectively.

Our proposed model can be overviewed by the block diagram, as shown in Figure 4. The network consists of two major paths, one to produce the contrast image and the other to predict the background coefficient. The contrast images should contain values of coefficients relative to its background coefficients ($\mu_{a}$,$\;\mu_{s}^{\prime }$). Then, output images are obtained by multiplying each image with its background coefficient. The reason for separating outputs into coefficients and contrasts is because of the difference of the known range between $\mu_{a}$ and $\mu_{s}^{\prime }$. Contrast images have the values ranged between 0 and 1, so it is straightforward to calculate the loss metrics. We then decided the architecture was to be consists of two separated paths between coefficients and contrast images, only to be multiplied to give the final outputs of distribution of coefficient values. We think that this separation-of-work strategy will improve training convergence, since each path can be treated as its own network, therefore, functionally independent between one and the other.

We define blocks specified to handle the input data. We want to convert from input data to a compressed space first to make sure the number of trainable parameters are not too huge. From Figure 4 we can see that the main network is the background predictor for the coefficients and domain-transform for the contrasts. U-net serves to improve the resulted contrast images, analogously with CNN after firstly the FCNN in previous studies.

**Figure 5:**
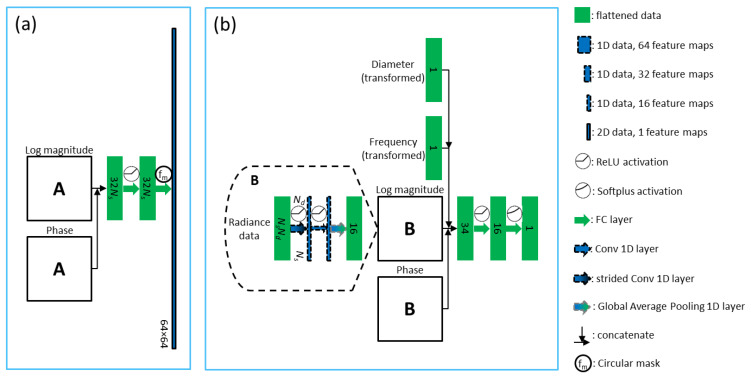
Proposed deep learning model including (**a**) domain-transform and (**b**) background predictor.

**Figure 6:**
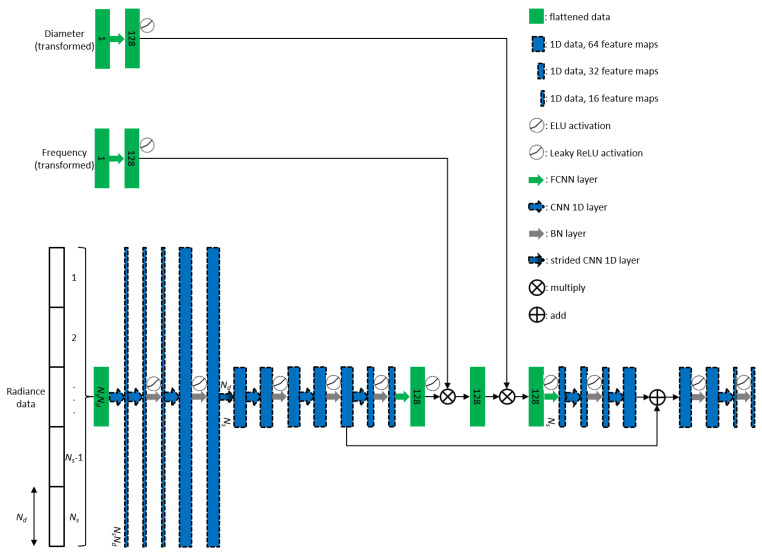
Network architecture of the block A.

The concept of encoder-decoder is extensively used [22], resulting with overall deep-layered network. Figure 5 illustrates the implementation of domain-transform and background predictor in the deep learning network. In Figure 5b, block B consists of two 1D convolutional layers followed by a global average pooling layer, as seen in Figure 5. ReLu activation was used after each convolutional layer. So, we already use a convolutional layer for spatial data that is not 2D (image). It has less trainable parameters and is able to create feature maps [22]. The first layer has a stride equal to the number of detectors (*N_d_*), since we have source-detector pair of measurement data, the result will be 1D data equal to the number of sources. Conceptually, every data-point from a single source is “collected” to be one data-point and converted to feature maps, because the smallest independent experiment is from one source activation. Each set of *N_d_* data points that belong to a source location has similar shape. By using the strided convolutional layer, significant features are extracted from these distinctive detection curves and are combined for all light sources. This idea is also used similarly for block A (see Figure 6). The second layer produces 16 feature maps, so each feature map is averaged at the spooling layer, resulting output neurons of size 16. We simply concatenate the resulted alternative-space data from block B with additional inputs such as diameter of phantom and frequency used for the experiment. Two fully connected layers added before the output resulted, with softplus activation in the end. It gives a nonzero output value with the advantage of smoothing, particularly useful for obtaining stable convergence [23].

In Figure 6, block A is a deep-layered network consist of 23 layers. Two convolutional layers followed by batch normalization [24] and ELU activation [25] employed before the size of the feature maps changed from 16 to 64 by the next convolutional layer, and then followed by another batch normalization and leaky ReLu activation [26]. ELU activation was used initially because it has the advantage of zero-mean activations and noise-robust deactivation. This will reshape the distribution of the noisy input signals. At this point, the input dimension has not changed. Then, after strided the convolutional layer in similar fashion with block B, the other series of layers was added. In the middle, however, we also added encoder-decoder structure with skip connection. Deep convolutional layers with residual learning [27], batch normalization layers and leaky ReLU activations to address the commonly known vanishing gradient problem [28], and to accelerate the training process. Additional inputs are converted into alternative-space data of size 128 before incorporated to bottleneck layers of this structure by multiplication. This block produces 16 × number of sources data. Followed by two fully connected layers with circular mask in the end, the domain-transform network produces initial image data before fed to U-net. The reason for built deeper network is that we want to improve the performance of the model for noisy data as well.

The U-net [2] to extract the contrast features of the image was implemented, though it is different from the original paper for the amount of layers and dimensions. Moreover, skip connections were used by adding the input and the output to make the network behave as residual learning. Absorption and reduced scattering coefficients were treated as different channels in a single image to be processed in U-net. Finally, the contrast images from the network can be obtained.

We propose a deep learning model as an alternative method capable for DOT image reconstruction by employing multiple deep learning architectures in imaging fields. Many structures such as encoder-decoder, skip connections, and U-net [2] were involved for the implementation. The branching-like overall structure and the specialized role of blocks divided for all inputs and outputs made it different from other existing networks. This model will be fully compatible for the considered ring-scanning measurement system. We design the model to be reliable and robust with any discrepancy in measurement data, by considering all of the inputs and outputs that can be provided and also expect noisy data, we are avoiding reuse and retrain the model each time for a specific case of different kinds of dataset. Thus, it is one-time for all solutions.

### 2.4. Training and Testing Environment

**Figure 7:**
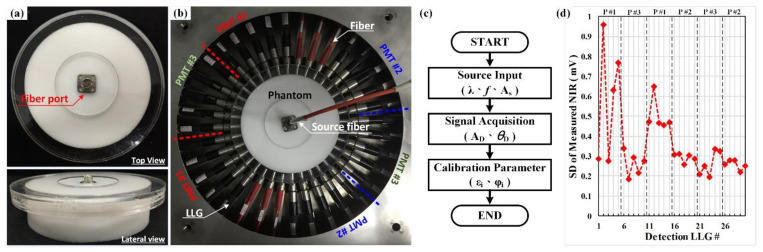
Measurement calibration: (**a**) calibration phantom (top and lateral views); (**b**) 3D scanning system with the calibration phantom; (**c**) flowchart of calibration procedure; and (**d**) standard deviations of measured data in mV for 30 detections [32].

Training and validation datasets obtained from the inhouse MATLAB^®^-coded TR computation scheme, NIR•FD_PC [29,30,31], were employed to create image-reconstruction deep-learning models. It is noted all the simulation samples were prepared by our inhouse computation code NIR•FD_PC [29,30,31], and the system calibration for both experimentation and computation scheme was performed on ring scanning rotating test bench [32,33,34], as shown in Figure 7. The datasets with 16 × 15 measuring points (amplitude and phase shift) of each has been employed to perform DOI from laboratory experiments at our institute. We performed scanning and image reconstruction for cylindrical phantoms with inclusion(s) to verify the performance of the imaging system. To calibrate the measurement module of the imaging system, especially for detection fibers and PMTs, a homogeneous cylindrical calibration phantom was employed [32]. The calibration phantom was made of silicone as a matrix and mixed with carbon and TiO_2_ powders for adjusting the absorption and scattering properties. The measurements were taken on solid phantoms.

The average computation time for the TR method to obtain a sample of the training dataset is around 154 s. Meanwhile, the training, validation and testing of the developed deep neural network computation algorithm were executed in Python using the Keras library. Table 3 specifies the computation environment of the implemented deep neural networks. To evaluate their image reconstruction performance, a measure to the errors of reconstructed optical coefficients, i.e., the mean squared error (MSE), was defined below

(3) $$\mathrm{MSE}\left(\mu \right)=\frac{\sum_{i=1}^{N}{\left(X_{i}-X_{i}^{true}\right)}^{2}}{N},$$

where μ means a specific optical property regarding background or contrast image; N denotes the number of voxels (data points considered inside the output grid); $X_{i}$ and $X_{i}^{true}$ are the reconstructed and the actual designated values, respectively. It is noted the final evaluated optical-property images for the absorption ($\mu_{a}$) and reduced scattering ($\mu_{s}^{\prime }$) coefficients are $c_{a}\mu_{0a}$, and $c_{s}\mu_{0s}^{\prime }$, where $c_{a}$, $c_{s}$, $\mu_{0a}$, and $\mu_{0s}^{\prime }$ denote the absorption contrast image, reduced scattering contrast image, background absorption coefficient image, and background reduced scattering coefficient image, respectively. Further, in order to give an overall assessment for the evaluated optical-property images, both $\mu_{a}$ and $\mu_{s}^{\prime }$, we here proposed a customized loss function of target *X*, *Q(X)*, defined by a weighted sum of varied MSE(*μ*) from each reconstructed image, i.e.,

(4) $$Q\left(X\right)=w_{im_{a}}\mathrm{MSE}\left(c_{a}\right)+w_{im_{s}^{\prime }}\mathrm{MSE}\left(c_{s}\right)+w_{a}\mathrm{MSE}\left(\mu_{0a}\right)+w_{s}^{\prime }\mathrm{MSE}\left(\mu_{0s}^{\prime }\right),$$

where $w_{im_{a}}$, $w_{im_{s}^{\prime }}$, $w_{a}$, and $w_{s}^{\prime }$ are the corresponding weights of each MSE(μ). In this way, relative errors with using contrast images instead of actual coefficient images are obtained so as to correctly judge the quality of reconstructed images since the images characterize the magnitude differences of optical-property coefficients between tumor (or inclusion) and background tissue. Additionally, the errors of background coefficients are also added up so that one can judge the ability of the computation model to evaluate the background coefficients. These weights were fixed as the parameters defined during training, and they were chosen manually. The basic idea was to balance each of the contributing losses to the overall assessment since the contrast images contribute more to the losses. We intended to include losses from the background contrast in order to be contributing more to the loss function, as opposed to calculate loss directly from output coefficient images. Thus, we chose $w_{im_{a}}$, $w_{im_{s}^{\prime }}$, $w_{s}$ equal to 1 while $w_{a}=100^{2}$ to balance the loss value with $w_{s}$, since the values differ around 100 times (see Table 2). The mean squared errors were calculated so as to choose $w_{a}$ simply $100^{2}$ and $w_{s}$ to be 1.

**Figure 8:**
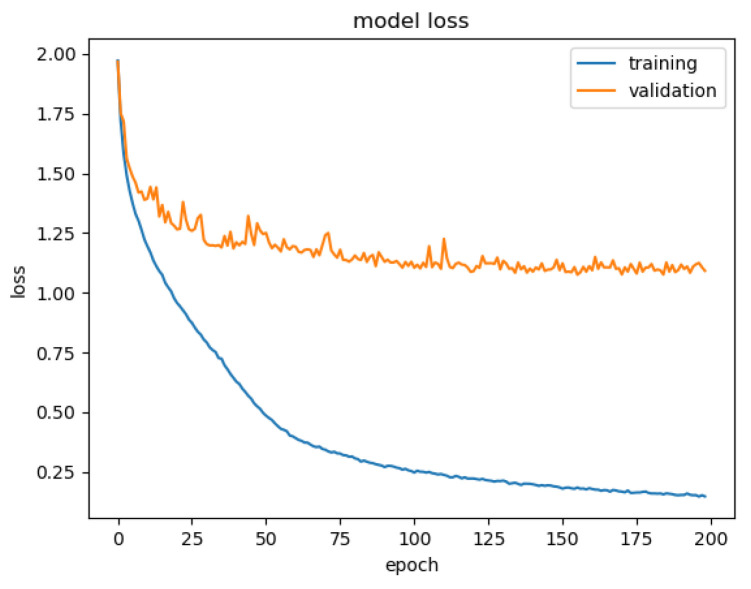
Loss during training and validation phases along with iteration epochs.

In total, it took 21.6 h for 200 epochs to achieve the trained model. Figure 8 illustrates the loss during training and validation with the datasets. It is found that the convergence for validation loss is different from training loss. This may be caused by the additional noises incorporated in the dataset.

## 3. Results

### 3.1. Test of Trained Models

**Figure 9:**
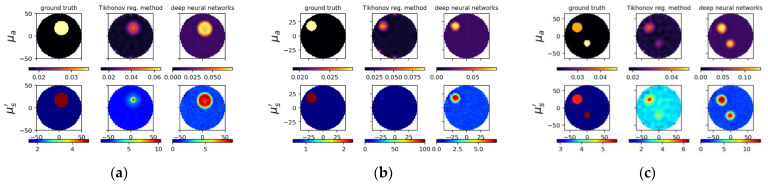

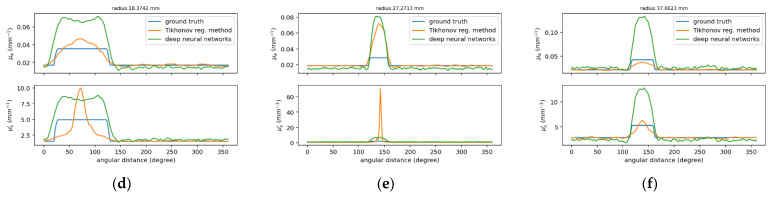
Reconstructed images for simulated cases A1675, A4144 and A6392 from left to right, respectively. (**a**–**c**) Designated and computed optical-property images, (**upper**) *μ_a_* and (**lower**) $\mu_{s}^{\prime }$ image; in (**a**–**c**), (**left**) ground truth, and reconstructed images using (**middle**) TR and (**right**) deep neural networks. (**d**–**f**) Circular cross-section profile of (**upper**) *μ_a_* and (**lower**) $\mu_{s}^{\prime }$ distribution that intersects with an (outer) inclusion.

**Figure 10:**
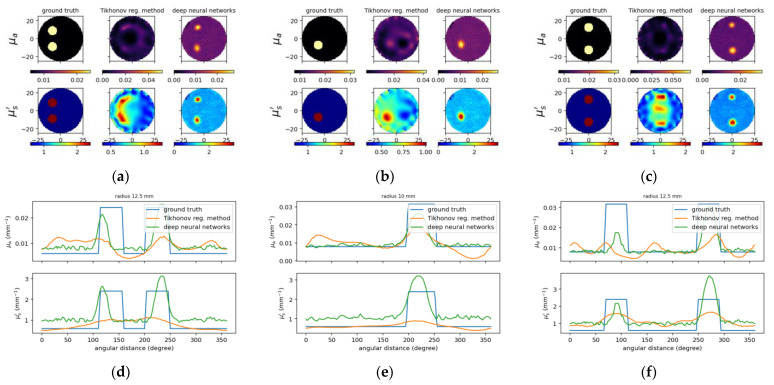
Reconstructed images for experimental case B2, B3 and B7 from left to right, respectively. (**a**–**c**) Designated and computed optical-property images, (**upper**) *μ_a_* and (**lower**) $\mu_{s}^{\prime }$ image; in (**a**–**c**), (**left**) ground truth, and reconstructed images using (**middle**) TR and (**right**) deep neural networks. (**d**–**f**) Circular cross-section profile of (**upper**) *μ_a_* and (**lower**) $\mu_{s}^{\prime }$ distribution that intersects with the center of inclusions.

We trained the model on 10,000 synthesized samples of simulation dataset and then tested on 10 samples of experimental dataset. The 10,000 samples consisted of 8000 samples for the training set and 2000 samples for the validation set (see Table 2). We list the phantom parameters for 10 simulation examples from the 10,000 samples as examples (Table 4) and all 10 samples from the experimental dataset (Table 5), respectively. Figure 9 and Figure 10 illustrate the reconstructed images through using the trained model and the TR method, as well as the associated cross-section profile of $\mu_{a}$ and $\mu_{s}^{\prime }$ images. Generally, the proposed model can successfully localize the inclusions in spite of inaccuracies. It is noted that probable phantom fabrication errors may exist for the experimental testing. The results prove that the proposed model is capable to perform better than the TR method. We can observe that the results from proposed deep learning model are smoother compared to the TR method. Moreover, no matter the size and contrast of the inclusion they do not matter for the deep learning model because it can still perform steadily, while these will affect the result for the TR method. Note that we also included two inclusions in these examples, and it has still shown similar performances. It is, however, found that the overestimation tendency occurs among the results from the proposed deep neural networks, as will be discussed later.

**Figure 11:**
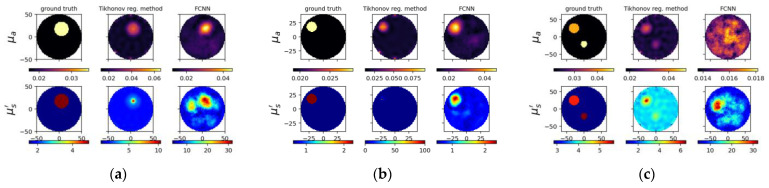

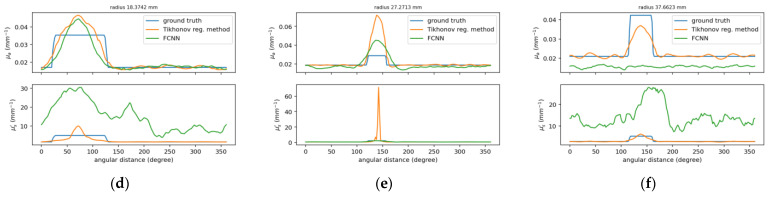
The same caption as in Figure 9 except the reconstructed images (**a**–**c**) on the (**right**) using FCNN and one of circular cross-section profiles using FCNN (**d**–**f**).

**Figure 12:**
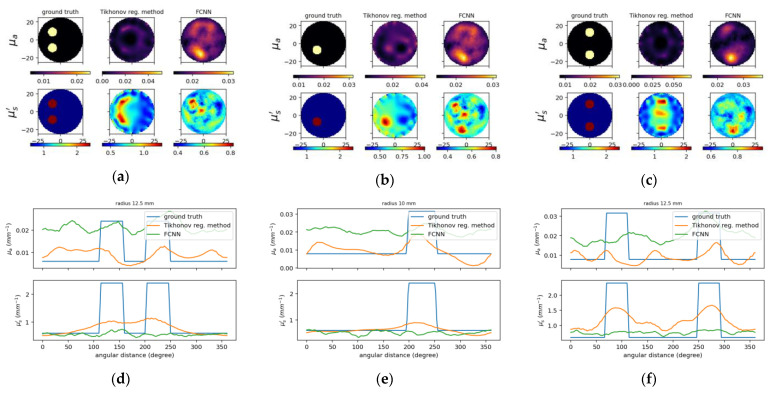
The same caption as in Figure 10 except the reconstructed images (**a**–**c**) on the (**right**) using FCNN and one of circular cross-section profiles using FCNN (**d**–**f**).

To compare the results between the proposed deep neural network and the deep network with fully-connected layers, Figure 11 and Figure 12 show the reconstructed optical properties and circular cross-section profiles through inclusion(s) for simulated and experimental cases, respectively. One may find by comparing Figure 10a–c with Figure 12a–c that the proposed NN model can reconstruct images from experimental data, while FCNN failed to detect the inclusions.

### 3.2. Performance Analysis

In order to assess the reconstructed images quantitatively, two measures (contrast and size resolution) were evaluated over the region of interest. The concept of definition of the contrast and size resolution originates from the precision and density/saturation conceptually, respectively, of which the advantage is to be implemented easily. The contrast resolution $R_{cont.}^{2D}$ is defined to evaluate the resolution on the contrast of optical property values of the inclusion relative to the background, i.e., [31,35]

(5) $$Ro_{cont.}^{2D}=\frac{{\left({\bar{\max}}^{incl.}/{\bar{\min}}^{back.}\right)}_{Recon.}}{{\left({\bar{\max}}^{incl.}/{\bar{\min}}^{back.}\right)}_{Orig.}},$$

and

(6) $$R_{cont.}^{2D}=\{\begin{array}{c} 2-Ro_{cont.}^{2D},\;\mathrm{if}\;R_{cont.}^{2D}>1\\ Ro_{cont.}^{2D},\;\mathrm{otherwise} \end{array},$$

where $\bar{\max}$ and $\bar{\min}$ denote the average of maxima and minima over all the selected inclusion regions, due to the possibility of some oscillations in these regions. This measure for the contrast resolution is designed to make the value of 1.0 that indicates obtaining the best precise contrast estimation of a reconstructed image. The value of the evaluation between 0 and 2.0 shows a little underestimation (if smaller than 1.0) or overestimation (if larger than 1.0). The definition in Equation (6) for ($2-Ro_{cont.}^{2D}$) gives this idea. Further, a negative value of $R_{cont.}^{2D}$ from Equation (6) means an overestimation occurs. To match the definition of contrast to assess the visibility of a structure in an image [36], Equation (5) is further modified as [37]

(7) $$Ro_{cont.}^{2D}=\frac{{\left(\left({\bar{\max}}^{incl.}-{\bar{\min}}^{back.}\right)/{\bar{\min}}^{back.}\right)}_{Recon.}}{{\left(\left({\bar{\max}}^{incl.}-{\bar{\min}}^{back.}\right)/{\bar{\min}}^{back.}\right)}_{Orig.}}\equiv \frac{{\left(\Delta I/\langle I\rangle \right)}_{Recon.}}{{\left(\Delta I/\langle I\rangle \right)}_{Orig.}}\equiv \frac{C_{Recon.}}{C_{Orig.}}$$

where C and I denote contrast and intensity, respectively. Moreover, to avoid probable outlier values that act as noise in images, the percentile values instead were employed; for instance, $\bar{\max}$ and $\bar{\min}$ were replaced by the 90th and the 10th percentiles. Additionally, the size resolution was defined as [31,35]

(8) $$R_{size}^{2D}=\sqrt{\left\{\left[1-\frac{{\left({\mathrm{RMSE}}^{incl.}\right)}_{Recon.2Orig.}}{{\left({\mathrm{RMSE}}^{incl.}\right)}_{Orig.2base.}}\right]R_{cont.}^{2D}\right\}}\equiv \sqrt{Ro_{size}^{2D}\cdot R_{cont.}^{2D}}$$

to evaluate the resolution over all of the inclusion size, where the RMSE (root mean squared error) was calculated over the whole 2D image domain or the region of interest, between the original (designated) value of inclusions and the baseline (reconstructed) value. The baseline values were used the same as the background optical coefficients. It should be noted that in order to prevent size overestimation the size resolution, Equation (8), includes the contrast resolution.

Following the above two measures on contrast and size, we integrated them into one for the numerical contrast-and-size detail (CSD) analysis to evaluate the performance of the reconstruction algorithms by defining [35]

(9) $$R_{contrast-size-detail}^{2D}=\sqrt{R_{cont.}^{2D}R_{size}^{2D}}.$$

The CSD is used to cope with the drawbacks of only contrast-detail analysis. The integrated contrast and size resolution is to evaluate both the contrast precision and the size accuracy for the image reconstruction scheme. Note that the measure defined by Equation (9) associated with Equations (5)–(8) emphasizes more on the accuracy to prevent overestimation.

After evaluating the reconstructed images, we applied the CSD analysis to both the simulated and experimental datasets, each with 10 samples, and compared the performance between the TR method and the trained deep learning model. We already define the contrast, size, and, CSD resolution. For the contrast resolution, Equation (5) was judged as

(10) $$Ro_{cont.}^{2D}\{\begin{array}{c} >\frac{1}{c},\;\mathrm{normal}\\ =\frac{1}{c},\;\mathrm{no}\;\mathrm{contrast}\\ <\frac{1}{c},\;\mathrm{abnormal} \end{array}$$

where c=8 is the maximum contrast and the abnormal situation means the optical value of the inclusion is smaller than that of the separation region. For the size resolution, the values of $Ro_{size}^{2D}$ are always less than unit and negative exhibits high underestimation or overestimation.

Table 6, Table 7, Table 8 and Table 9 list the contrast, size, and CSD resolutions. The coloring in Table 6, Table 7, Table 8 and Table 9 is to help understand how well the reconstruction methods perform to recover the images, especially it is helpful for the fewer experimental samples. The reference value (here T = 0.3) is a designated measure which says 30% of the original contrast and/or size of the ground truth can be recovered. ‘Green’ values indicate good resolution among the test samples. ‘Yellow’ values indicate fair resolution and mean fulfilling the chosen reference T = 0.3. ‘Red’ values indicate relatively bad resolutions. The other values left uncolored mean that their resolutions cannot meet the specified criterion. Some interesting results can be observed that, in Table 7, many samples from the simulation dataset reconstructed with the proposed method cannot pass the criterion. These actually result from the overestimation. Later, further analysis was performed on the issue of overestimation. For experimental samples, most of the resolution values from proposed method fulfill the criterion. They are comparable with the TR results.

**Figure 13:**
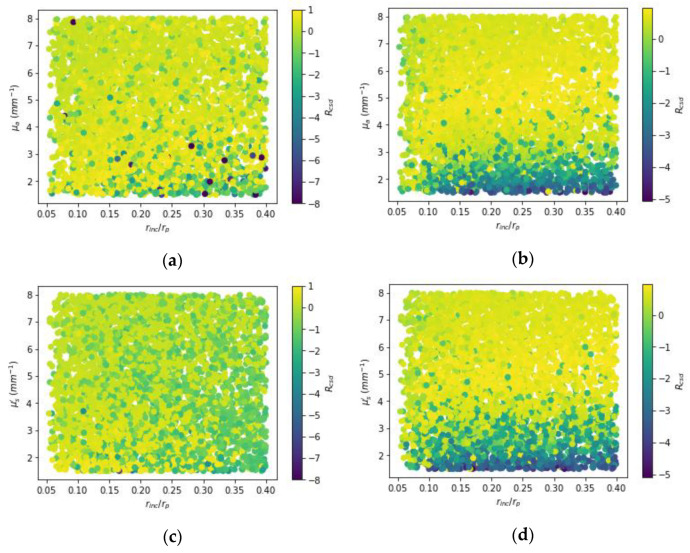
Scatter plots of the CSD resolution for one-inclusion samples. (**a**,**b**) show the resolutions for absorption (*μ_a_*) while (**c**,**d**) for reduced scattering ($\mu_{s}^{\prime }$). Besides, on the left (**a**,**c**) from Tikhonov regularization while on the right (**b**,**d**) from proposed model.

Figure 13 shows the scatter plots that illustrate the distribution of CSD resolution vs. optical-property contrasts and relative size of inclusions for varied methods. Here, one-inclusion samples with a total amount of 4400 cases were taken to investigate the relationship. For the proposed method, the trend was towards negative values, especially, for the low contrasts, indicating an overestimation tendency. It is observed for the low contrasts between 1.5 and 2.5 highly negative resolutions frequently occur; besides, for the contrasts above 4 much better CSD resolutions can be obtained. As to the TR method, larger inclusions yield the CSD resolutions toward negative values due to the TR being unable to recover all the areas of the inclusions with larger size, only for the most part while the magnitude would be dampened.

**Figure 14:**
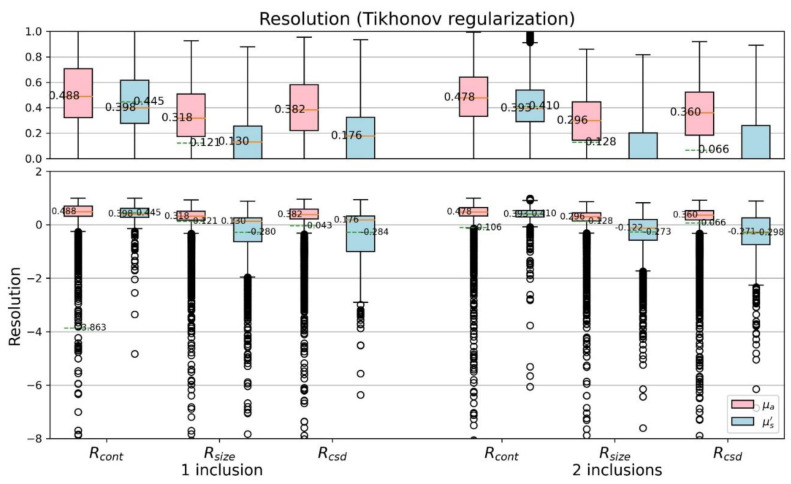
Boxplots of CSD resolution (TR).

**Figure 15:**
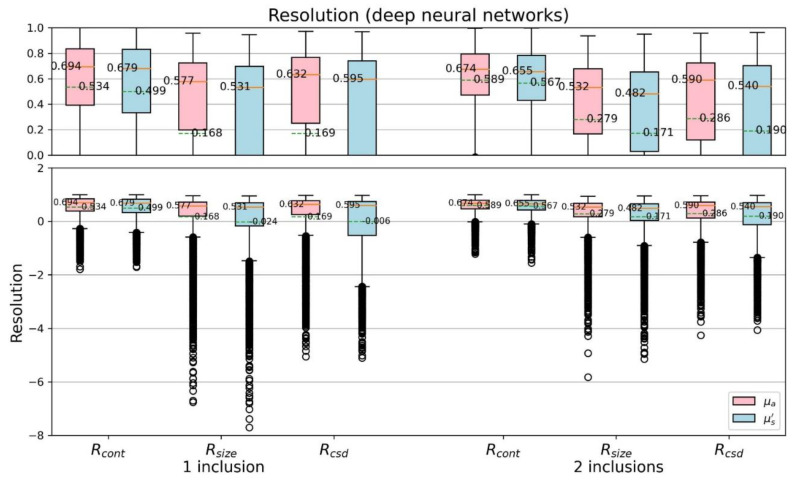
Boxplots of CSD resolution (proposed model).

Figure 14 and Figure 15 further discuss the CSD resolutions, shown with boxplots. We can find, especially by the median and mean value, that the proposed model is overall better than the TR method, no matter they are one- or two-inclusion phantom cases. Both the contrast resolution (Equation (7)) and the size resolution (Equation (8)) are important to contribute the CSD resolution (Equation (9)). The results reveal the CSD analysis suitable for quantitatively assess the performance of varied reconstruction methods.

**Figure 16:**
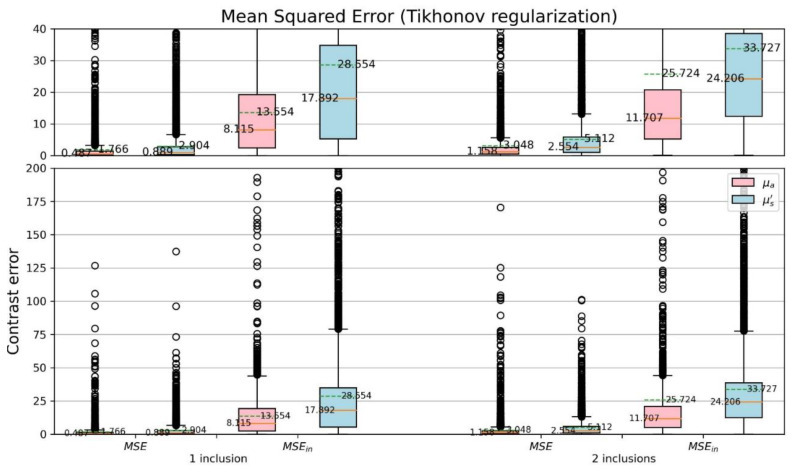
Boxplots of MSE for $\frac{\mu }{\mu_{base}}$ (TR).

**Figure 17:**
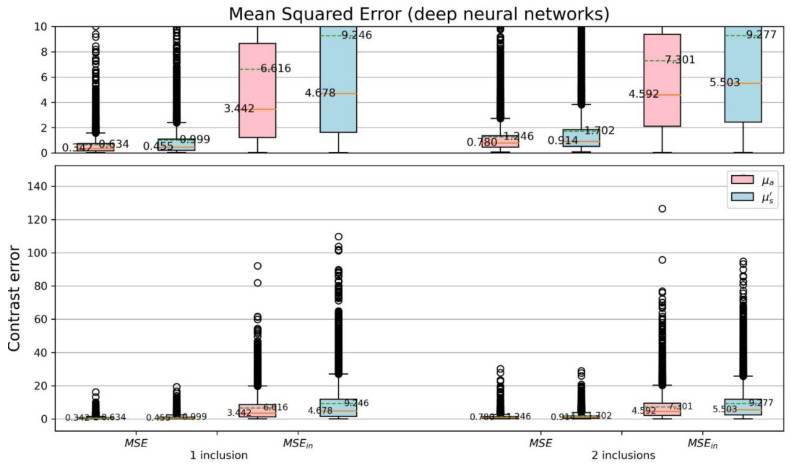
Boxplots of MSE for $\frac{\mu }{\mu_{base}}$ (proposed model).

For more detail of the CSD analysis, the mean squared errors (MSEs) of reconstructed optical-property contrasts for the inclusion to the background of phantom were calculated (Figure 16 and Figure 17). To calculate relative errors is more significant as each sample has its specified background coefficients. The detection of inclusions in a sample depends on the reconstructed contrast. Thus, a dimensionless quantity $\frac{\mu }{\mu_{base}}$ was used, where μ denotes either original ($\mu_{Orig.}$) or reconstructed ($\mu_{Recon.}$) coefficient, and $\mu_{base}$ is the background coefficient ($\mu_{Orig.}^{back.}$) of phantom. The MSE and $MSE_{in}$ were evaluated, where $MSE_{in}$ is the mean squared error considering only the area of inclusions for each sample. Comparing Figure 16 with Figure 17 the proposed network yields smaller errors than the TR method does.

## 4. Discussion

A deep convolutional neural network model that employs blocks of decoders and fuses multiple architectures has been implemented and trained successfully. The proposed model generally tends to overestimate the coefficient values, indicated by high negative values of the resolutions present. However, overall it shows that the proposed model is capable to handle various input data. From the simulation dataset, we found that the proposed model is feasible as an alternative to the TR method, indicated by its localization capability. This remains consistent for experimental samples as well.

Note that the weights used for testing the model are selected manually from the seventh epoch of the training process instead of the last epoch. This means that overfitting still occurs when we tried to test using experimental data. There does exist some discrepancy between training and validation samples after a few iterations. For further development of the algorithm, one might consider an attempt for regularization, or use more training data.

The overestimation tendency occurred in the proposed model. From Figure 9 it is found that the sectional profile looks exactly similar between absorption and scattering coefficient. This may indicate that the two images only differ by scale, which is undesirable since the contrast can be different between absorption and scattering coefficient. One major reason is due to the CNN architecture. Since two images stack and go through a single path of CNN, the contour become exactly similar for both images. The separate into two different paths could be helpful to improve the image reconstruction.

## 5. Conclusions

In this study, a deep learning CNN model as an alternative to the existing TR method for diffuse optical imaging was proposed and implemented. The training dataset consisting of 10,000 samples with different designs of phantom cases was prepared. The parameters of these phantom cases were specified based on various properties. For each sample, the input data are in the form of 16 × 15 × 2 values (16 source/detector locations), which are log amplitude and phase delay. The output data are in the form of a rectangular grid of size 64 × 64, each for both absorption and scattering coefficients. These values have been interpolated from the original data of 3169 nodes. The architecture of network model was designed based on varied ideas. The transformation from sensor-domain to image-domain, and an encoder to learn a compressed representation of the inputs were applied. After the compression and transformation of the inputs to the image domain, the U-net with skip connections is used to extract features to obtain a contrast image. The constructed images are finally obtained by multiplying the contrast image with background coefficients.

The custom loss function was defined by the sum of the weighted MSE of computed contrast image and background coefficients. In the training phase, the iteration was proceeded to minimize the loss; additionally, Adam optimizer with β_1_ = 0.5, a learning rate of 0.0002 and a batch size of 32, were employed. The model was trained using 21.6 h for 200 iteration epochs. As the training loss drops quickly after only a few iterations, thus the architecture of deep learning network is considered ideal. A further generalization can still be performed so as not to overfit the model. For testing the trained model, both simulated- and experimental-sample datasets were employed to examine the performance of image reconstruction. The main advantage of this computation scheme is its superior efficiency against the conventional FEM-based image reconstruction method. Especially, the contribution of this study is the first proposed deep CNN model for DOT image reconstruction by combining multiple state-of-the-art deep learning architectures in imaging fields to have superior performance to learn than the FCNN and/or CNN. Though the calibration for software and hardware is a kind of system dependent, the constructed and trained network can be applied for other calibrated experimental system since one can obtain the acquired data with the associated quantities and physical units. Certainly, the sectional profiles are not always only circular, such as oval from clinical case examples. In the field of deep learning the reconstruction of optical-property images with other shapes of profile is an issue of transfer learning. More specifically, the trained network based on huge circular-profile data can be adapted to a new network with using a relatively small amount of oval-profile data (or other shapes) by a transfer learning technique.

## Figures and Tables

**Table 1:** Summary of related works.

Specifications	Feng et al. [14]	Yedder et al. [15]	Yoo et al. [16]
Measurement system	FD	CW	FD (3-D)
S × D	16 × 15	2 × 128	64 × 40
Input	240	256	2560
Training	20,000	4500	1000
Validation	1045	200	500
Epoch	16,000	2000	120
Trainable parameters	1,560,191	>4,000,000	137,625,600
Output	2001	128 × 128	32 × 64 × 20
Network type	FCNN	FCNN + CNN	FCNN + CNN

**Table 2:** Parameters of the training datasets.

Parameters	Range or Value
2D shape and size	Circular, 60–150 mm in diameter
# of sources/detectors	16
Frequency	10–100 MHz
FEM mesh	3169 nodes and 6144 elements
Background absorption coefficient	0.005–0.03 mm^−1^
Background scattering coefficient	0.5–3 mm^−1^
contrast of inclusion to background	1.5–8
Inclusion radius	4–17 mm
Partition (based on # of inclusions)	0: 1%, 1: 44%, 2: 55%
Partition (training and validation)	Training: 80%, Validation: 20%
Total training and validation samples	10,000

**Table 3:** Specification and computation environment of the implemented deep neural networks.

Specifications	Information
Loss function	Weighted sum of MSE
Optimizer	Adam (β_1_ = 0.5)
Learning rate	0.0002
Batch size	32
Epochs	200
Framework	Keras with Tensorflow backend
Environment	JupyterLab
GPU	NVIDIA GeForce GT 640 (2 GB memory)
CPU	Intel Core i7-5960X 3.00 GHz
RAM	24 GB

**Table 4:** Phantom parameters of simulated samples for training or validation, where the phantom cases in the gray area possess two inclusions.

CASE	*D* (mm)	*f* (MHz)	*μ_a_* (mm^−1^)	$\mu_{s}^{\prime }$ (mm^−1^)	*r* (mm)	*r_oc_* (mm)	*θ_oc_* (°)	*c_a_*	$c_{s}^{\prime }$
A466 (validation)	110	0	0.0079	2.75	17.14	22.94	104.78	1.52	1.56
A1675 (training)	100	100	0.0170	1.52	15.91	18.37	72.69	2.08	3.25
A3381 (training)	130	10	0.0157	1.53	4.75	39.72	352.69	1.78	3.30
A4144 (training)	80	20	0.0189	0.66	8.95	27.27	138.73	1.53	3.40
A4483 (validation)	60	60	0.0103	0.71	4.05	7.11	260.35	2.06	3.44
A4617 (training)	100	100	0.0150	0.90	[14, 11]	[13, 34]	[255, 225]	[2, 2]	[3, 2]
A5741 (training)	130	100	0.0115	1.25	[8.63, 4.15]	[46.39, 51.55]	[118.06, 55.02]	[2.36, 2.155]	[3.31, 2.34]
A6392 (training)	130	60	0.0210	2.86	[14.36, 9.28]	[37.66, 20.85]	[139.07, 270.32]	[2.02, 2.25]	[1.86, 1.98]
A6472 (validation)	90	40	0.0221	1.25	[9.86, 6.49]	[33.94, 27.37]	[21.20, 18.47]	[2.27, 2.29]	[2.31, 2.23]
A8223 (training)	70	70	0.0156	0.90	[13.69, 5.64]	[17.50, 20.16]	[152.47, 138.59]	[2.16, 2.33]	[3.36, 1.64]

**Table 5:** Phantom parameters of experimental samples for testing, where the phantom cases in the gray area possess two inclusions.

CASE	*D* (mm)	*f* (MHz)	*μ_a_* (mm^−1^)	$\mu_{s}^{\prime }$ (mm^−1^)	*r* (mm)	*r_oc_* (mm)	*θ_oc_* (°)	*c_a_*	$c_{s}^{\prime }$
B1	50	20	0.006	0.6	5	12.5	180	4	4
B2	50	20	0.006	0.6	[5, 5]	[12.5, 12.5]	[225, 135]	[4, 4]	[4, 4]
B3	50	20	0.0079	0.6	5	10	225	4	4
B4	50	20	0.006	0.6	5	10	180	4	4
B5	50	20	0.0079	0.6	5	12.5	180	4	4
B6	50	20	0.0079	0.6	5	12.5	180	4	4
B7	50	20	0.0079	0.6	[5, 5]	[12.5, 12.5]	[90, 270]	[4, 4]	[4, 4]
B8	50	20	0.0079	0.6	5	12.5	180	4	4
B9	50	20	0.0079	0.6	5	12.5	180	4	4
B10	50	20	0.006	0.6	5	12.5	180	4	4

**Table 6:** Contrast, size, and CSD resolution (TR for simulated samples).

CASE	$R_{cont.}^{2D}$		$Ro_{size}^{2D}$		$R_{size}^{2D}$		$R_{csd}^{2D}$	
	$\mu_{a}$	$\mu_{s}^{\prime }$	$\mu_{a}$	$\mu_{s}^{\prime }$	$\mu_{a}$	$\mu_{s}^{\prime }$	$\mu_{a}$	$\mu_{s}^{\prime }$
A466	0.63	0.87	0.06	0.39	0.19	0.58	0.35	0.71
A1675	0.88	0.60	0.62	0.28	0.74	0.41	0.81	0.50
A3381	0.85	0.38	0.46	−0.04	0.63	−0.26	0.73	−0.65
A4144	−0.02	0.74	−1.40	−4.50	−1.68	−2.38	−1.84	−1.74
A4483	0.98	0.35	0.78	0.08	0.87	0.17	0.92	0.25
A4617	0.85	0.86	0.40	0.25	0.56	0.46	0.68	0.63
A5741	0.81	0.56	0.51	0.23	0.64	0.36	0.72	0.45
A6392	0.69	0.78	0.36	0.36	0.49	0.53	0.58	0.64
A6472	0.68	0.79	0.19	−0.89	0.36	−0.52	0.50	−0.36
A8223	−0.14	0.51	−0.85	−5.92	−1.34	−2.85	−1.69	−2.04

**Table 7:** Contrast, size, and CSD resolution (proposed model for simulated samples).

CASE	$R_{cont.}^{2D}$		$Ro_{size}^{2D}$		$R_{size}^{2D}$		$R_{csd}^{2D}$	
	$\mu_{a}$	$\mu_{s}^{\prime }$	$\mu_{a}$	$\mu_{a}$	$\mu_{s}^{\prime }$	$\mu_{a}$	$\mu_{s}^{\prime }$	$\mu_{a}$
A466	−1.11	−1.05	−11.14	−0.50	−5.89	−1.23	−4.28	−1.94
A1675	−0.47	0.42	−0.60	0.11	−1.22	0.22	−1.73	0.30
A3381	0.79	0.43	0.56	0.22	0.66	0.31	0.72	0.36
A4144	−1.04	0.63	−2.20	−0.86	−2.59	−1.09	−2.81	−1.22
A4483	0.52	0.88	0.25	0.48	0.36	0.65	0.44	0.76
A4617	−0.46	−0.05	−0.67	−2.69	−1.28	−2.31	−1.78	−2.17
A5741	0.32	0.57	0.10	0.17	−0.17	0.25	−0.38	0.35
A6392	−0.29	−0.55	−1.59	−0.81	−1.90	−1.43	−2.09	−1.91
A6472	−0.05	−0.06	−0.02	−1.37	−0.33	−1.67	−0.61	−1.85
A8223	−0.38	−0.44	−0.81	−5.22	−1.39	−3.38	−1.82	-2.82

**Table 8:** Contrast, size, and CSD resolution (TR for experimental data).

CASE	$R_{cont.}^{2D}$		$Ro_{size}^{2D}$		$R_{size}^{2D}$		$R_{csd}^{2D}$	
	$\mu_{a}$	$\mu_{s}^{\prime }$	$\mu_{a}$	$\mu_{a}$	$\mu_{s}^{\prime }$	$\mu_{a}$	$\mu_{s}^{\prime }$	$\mu_{a}$
B1	0.26	0.94	−0.17	0.61	−0.55	0.76	−0.98	0.85
B2	0.73	0.48	0.14	0.23	0.32	0.33	0.48	0.40
B3	0.58	0.41	0.23	0.13	0.37	0.23	0.46	0.31
B4	0.78	0.41	0.36	0.18	0.53	0.27	0.64	0.33
B5	0.23	0.76	−0.17	0.45	−0.56	0.59	−0.99	0.67
B6	0.31	0.51	−0.09	0.17	−0.39	0.30	−0.81	0.39
B7	0.64	0.65	0.04	0.50	0.04	0.57	−0.04	0.61
B8	0.93	0.33	0.63	0.07	0.77	0.15	0.84	0.22
B9	0.33	0.72	−0.13	0.54	−0.46	0.62	−0.88	0.67
B10	0.98	0.51	0.52	0.19	0.72	0.31	0.84	0.40

**Table 9:** Contrast, size, and CSD resolution (proposed model for experimental data).

CASE	$R_{cont.}^{2D}$		$Ro_{size}^{2D}$		$R_{size}^{2D}$		$R_{csd}^{2D}$	
	$\mu_{a}$	$\mu_{s}^{\prime }$	$\mu_{a}$	$\mu_{a}$	$\mu_{s}^{\prime }$	$\mu_{a}$	$\mu_{s}^{\prime }$	$\mu_{a}$
B1	0.69	0.69	0.45	0.50	0.55	0.59	0.62	0.64
B2	0.49	0.49	0.30	0.40	0.39	0.45	0.44	0.47
B3	0.65	0.65	0.34	0.59	0.47	0.62	0.55	0.63
B4	0.69	0.69	0.48	0.55	0.58	0.61	0.63	0.65
B5	0.44	0.44	0.16	0.41	0.26	0.43	0.34	0.43
B6	0.38	0.38	0.12	0.38	0.22	0.38	0.29	0.38
B7	0.64	0.64	0.28	0.46	0.42	0.54	0.52	0.59
B8	0.89	0.89	0.43	0.37	0.62	0.57	0.74	0.71
B9	0.72	0.72	0.31	0.46	0.48	0.58	0.59	0.64
B10	0.93	0.93	0.52	−1.84	0.69	−1.40	0.80	−1.22

## References

[1] Krizhevsky A., Sutskever I., Hinton G.E. (2017). ImageNet Classification with Deep Convolutional Neural Networks. Commun. ACM.

[2] Ronneberger O., Fischer P., Brox T. (2015). U-Net: Convolutional Networks for Biomedical Image Segmentation. Lecture Notes in Computer Science (Including Subseries Lecture Notes in Artificial Intelligence and Lecture Notes in Bioinformatics).

[3] Pelt D.M., Batenburg K.J. (2013). Fast Tomographic Reconstruction from Limited Data Using Artificial Neural Networks. IEEE Trans. Image Process..

[4] Wang S., Su Z., Ying L., Peng X., Zhu S., Liang F., Feng D., Liang D. (2016). Accelerating Magnetic Resonance Imaging via Deep Learning. Proc.-Int. Symp. Biomed. Imaging.

[5] Zhu B., Liu J.Z., Cauley S.F., Rosen B.R., Rosen M.S. (2018). Image Reconstruction by Domain-Transform Manifold Learning. Nature.

[6] Gibson A., Dehghani H. (2009). Diffuse Optical Imaging. Philos. Trans. R. Soc. A Math. Phys. Eng. Sci..

[7] Dehghani H., Eames M.E., Yalavarthy P.K., Davis S.C., Srinivasan S., Carpenter C.M., Pogue B.W., Paulsen K.D. (2009). Near Infrared Optical Tomography Using NIRFAST: Algorithm for Numerical Model and Image Reconstruction. Commun. Numer. Methods Eng..

[8] Durduran T., Choe R., Baker W.B., Yodh A.G. (2010). Diffuse Optics for Tissue Monitoring and Tomography. Rep. Prog. Phys..

[9] Dehghani H., Sri Nivasan S., Pogue B.W., Gibson A. (2009). Numerical Modelling and Image Reconstruction in Diffuse Optical Tomography. Philos. Trans. R. Soc. A Math. Phys. Eng. Sci..

[10] Arridge S.R., Schotland J.C. (2009). Optical Tomography: Forward and Inverse Problems. Inverse Probl..

[11] Cao N., Nehorai A., Jacobs M. (2007). Image Reconstruction for Diffuse Optical Tomography Using Sparsity Regularization and Expectation-Maximization Algorithm. Opt. Express.

[12] Zhao Q., Ji L., Jiang T. (2006). Improving Performance of Reflectance Diffuse Optical Imaging Using a Multicentered Mode. J. Biomed. Opt..

[13] Uludaǧ K., Steinbrink J., Villringer A., Obrig H. (2004). Separability and Cross Talk: Optimizing Dual Wavelength Combinations for near-Infrared Spectroscopy of the Adult Head. Neuroimage.

[14] Feng J., Sun Q., Li Z., Sun Z., Jia K. (2018). Back-Propagation Neural Network-Based Reconstruction Algorithm for Diffuse Optical Tomography. J. Biomed. Opt..

[15] Yedder H., BenTaieb A., Shokoufi M., Zahiremami A., Golnaraghi F., Hamarneh G., Knoll F., Maier A., Rueckert D. (2018). Deep Learning Based Image Reconstruction for Diffuse Optical Tomography. Machine Learning for Medical Image Reconstruction, MLMIR 2018.

[16] Yoo J., Sabir S., Heo D., Kim K.H., Wahab A., Choi Y., Lee S.I., Chae E.Y., Kim H.H., Bae Y.M. (2020). Deep Learning Diffuse Optical Tomography. IEEE Trans. Med. Imaging.

[17] Yu J.-M., Pan M.-C., Hsu Y.-F., Chen L.-Y., Pan M.-C. (2015). Design and implementation of three-dimensional ring-scanning equipment for optimized measurements of near- infrared diffuse optical breast imaging. Opt. Eng..

[18] Chen L.-Y., Pan M.-C., Yan C.-C., Pan M.-C. (2016). Wavelength Optimization Using Available Laser Diodes in Spectral Near-Infrared Optical Tomography. Appl. Opt..

[19] Gunther J.E., Lim E., Kim H.K., Flexman M., Brown M., Refrice S., Kalinsky K., Hershman D., Hielscher A.H. (2013). Using diffuse optical tomography to monitor tumor response to neoadjuvant chemotherapy in breast cancer patients. Proc. SPIE.

[20] Al Abdi R., Graber H.L., Xu Y., Barbour R.L. (2011). Optomechanical imaging system for breast cancer detection. J. Opt. Soc. Am. A.

[21] Li X., Xi L., Jiang R., Yao L., Jiang H. (2011). Integrated diffuse optical tomography and photoacoustic tomography: Phantom validations. Biomed. Opt. Express.

[22] Lucas A., Iliadis M., Molina R., Katsaggelos A.K. (2018). Using Deep Neural Networks for Inverse Problems in Imaging: Beyond Analytical Methods. IEEE Signal Process. Mag..

[23] Zheng H., Yang Z., Liu W., Liang J., Li Y. Improving Deep Neural Networks Using Softplus Units. Proceedings of the 2015 International Joint Conference on Neural Networks (IJCNN), IEEE.

[24] Ioffe S., Szegedy C. (2015). Batch Normalization: Accelerating Deep Network Training by Reducing Internal Covariate Shift. 32nd Int. Conf. Mach. Learn. ICML 2015.

[25] Clevert D.-A., Unterthiner T., Hochreiter S. Fast and Accurate Deep Network Learning by Exponential Linear Units (ELUs). Proceedings of the 4th International Conference on Learning Representations (Poster).

[26] Xu B., Wang N., Chen T., Li M. (2015). Empirical Evaluation of Rectified Activations in Convolutional Network. arXiv.

[27] He K., Zhang X., Ren S., Sun J. Deep Residual Learning for Image Recognition. Proceedings of the IEEE Computer Society Conference on Computer Vision and Pattern Recognition, IEEE.

[28] Glorot X., Bengio Y. (2010). Understanding the Difficulty of Training Deep Feedforward Neural Networks. J. Mach. Learn. Res..

[29] Chen L.-Y., Yu J.-M., Pan M.-C., Sun S.-Y., Chou C.-C., Pan M.-C. (2016). Comparisons of Diffuse Optical Imaging between Direct-Current and Amplitude-Modulation Instrumentations. Opt. Quant. Elect..

[30] Chen L.Y., Pan M.C., Pan M.C. (2013). Flexible near Infrared Diffuse Optical Tomography with Varied Weighting Functions of Edge-preserving Regularization. Applied Optics.

[31] Chen L.Y., Pan M.-C., Chen C.-H., Pan M.-C., Shyr Y.-M. (2008). Highly resolved diffuse tomography: A systematic approach using high-pass filtering for value-preserved images. J. Biomed. Opt..

[32] Yu J.M., Pan M.C., Chen L.Y., Pan M.C., Hsu Y.F. (2017). Phantom verification for a ring-scanning and prone diffuse optical imaging system. Optics Commun..

[33] Pan M.-C., Yu J.-M., Chen L.-Y., Liang Y.-T., Pan M.-C. (2019). Optical-property coefficient estimation of bulky medium in experiments with a succinctly analytical calculation. Opt. Quant. Elect..

[34] Pan M.-C., Chen C.-H., Pan M.-C., Shyr Y.-M. (2009). Near infrared tomographic system based on high angular resolution mechanism—Design, calibration, and performance. Measurement.

[35] Chen L.Y., Pan M.C., Pan M.C. (2013). Visualized Numerical Assessment for near Infrared Diffuse Optical Tomography with Contrast-and-Size Detail Analysis. Opt. Rev..

[36] Wang L.V., Wu H. (2007). Biomedical Optics: Principles and Imaging.

[37] Yuliansyah D.R. (2020). Diffuse Optical Imaging Using Deep Convolutional Neural Networks. Master’s Thesis.

